# The Role of the Cysteamine Dioxygenase (*ADO*) Gene in Atopic Dermatitis

**DOI:** 10.2340/actadv.v106.43770

**Published:** 2026-01-27

**Authors:** Sailan WANG, Raquel VAZ, Josefin LYSELL, Jesper EISFELDT, Pelin SAHLÉN, Samina ASAD, Carl-Fredrik WAHLGREN, Magnus NORDENSKJÖLD, Maria BRADLEY, Isabel TAPIA-PAEZ

**Affiliations:** 1 Division of Dermatology and Venereology, Department of Medicine, Solna, Karolinska Institutet, Stockholm; 2 Department of Molecular Medicine and Surgery, Karolinska Institutet, Stockholm; 3 Department of Dermatology and Venereology, Karolinska University Hospital, Stockholm; 4 Department of Clinical Genetics and Genomics, Karolinska University Hospital, Stockholm; 5 Science for Life Laboratory, School of Chemistry, Biotechnology and Health, Royal Institute of Technology, Stockholm, Sweden; 6 Department of Dermatology, The First Affiliated Hospital, Zhejiang University School of Medicine, Hangzhou, China

**Keywords:** atopic dermatitis, taurine, zebrafish, Capture Hi-C, cysteamine dioxygenase, HaCaT cells

## Abstract

Atopic dermatitis is a chronic inflammatory skin disorder influenced by genetic and environmental factors. A chromosome conformation capture study identified the cysteamine dioxygenase (*ADO*) gene as being associated with atopic dermatitis in differentiating keratinocytes. We aimed to evaluate the causal and pathophysiological roles of *ADO* in atopic dermatitis. This study utilized transcriptomic data and immunostaining techniques to analyse ADO expression. Human keratinocyte cell line (HaCat), and zebrafish models were employed to explore the functional role of *ADO*. RNA sequencing and immunostainings indicated higher ADO expression in lesional skin than in non-lesional skin in atopic dermatitis patients. Moreover, atopic dermatitis patients carrying the risk allele (C) exhibited increased levels of ADO in lesional skin. *In vivo*, zebrafish embryos with dysregulated *ADO* expression displayed impaired epidermal morphogenesis, particularly in their tails, along with increased neutrophil infiltration, indicating an inflammatory response. *In vitro*, alterations in *ADO* expression in HaCaT cells led to expression changes of proinflammatory cytokines and skin barrier markers. Further, both upregulation and downregulation of *ADO* were associated with enhanced reactive oxygen species production. These findings suggest that the *ADO* gene plays a critical role in maintaining skin homeostasis, and its dysregulation contributes to inflammation and compromised skin barrier function in the pathogenesis of atopic dermatitis.

## SIGNIFICANCE

Atopic dermatitis is a common chronic inflammatory skin disease with a strong genetic basis affecting millions of people worldwide. This study focuses on the *ADO* gene and reveals that abnormal *ADO* expression in atopic dermatitis patients impairs skin barrier function and triggers inflammation. We show that *ADO* influences epidermal differentiation, immune responses, and oxidative stress, postulating it as a key genetic factor in atopic dermatitis. These findings advance our understanding of atopic dermatitis causes and suggest *ADO* could serve as both a diagnostic marker and a target for future therapies.

Atopic dermatitis (AD) is a complex and chronic skin disorder characterized by inflammation, xerosis (dry skin), erythema (redness), and pruritic (itchy) rashes, typically affecting the flexural areas of the arms and legs (1). It usually manifests in early childhood, with a prevalence of 15–20% in children and up to 10% in adults (2). It is considered to be a multifactorial disease, affected by both environmental and genetic factors (1). AD is linked to innate and adaptive Th2-cell-derived cytokines, which, in addition to immunological effects, alter the growth and differentiation of epidermal keratinocytes (3).

Numerous genes associated with AD have been identified through genome-wide association studies (GWAS). These genes are implicated in systemic immune regulation, cutaneous inflammation, neuroimmune interactions, environmental sensing, and epidermal barrier dysfunction (1, 4). A GWAS involving more than 1 million patients identified 91 genetic loci associated with AD (5). The filaggrin (*FLG*) gene is the most significant risk gene for AD. *FLG* is located on chromosome 1q21 in a region important for epidermal differentiation and keratinocyte cornification (6). The loss-of-function mutations in *FLG* increase the risk for AD by approximately 3-fold in heterozygous carriers (1).

However, the genetic variants identified to date account for only a modest portion of the heritability. Thus far, most single-nucleotide polymorphisms (SNPs) associated with AD through GWAS (https://www.ebi.ac.uk/gwas/) (7) are located in intergenic or intragenic regions, complicating the identification of their true functional targets, and they are frequently found within enhancers. The 3D genome structure, with chromatin looping, brings promoters close to their enhancers leading to changes in gene expression. Capture Hi-C technology combines Hi-C with sequence capture methodologies to map chromatin loops involving promoters using probes targeting around 25,000 promoters in a single experiment. This approach also helps identify new susceptibility genes, for instance in keratinocytes. In our previous study, we found interactions between cysteamine dioxygenase (*ADO)* gene promoter and a significant GWAS variant associated with AD, rs10995251 (8) (*p* = 6 × 10^–20^), which highlighted *ADO* (9).

*ADO* encodes the enzyme 2-aminoethanethiol dioxygenase. This enzyme is involved in the metabolism of cysteamine, a compound known to mitigate airway hypersensitivity and prevent asthma development (10, 11). Further, the *ADO* gene is crucial for the endogenous synthesis of hypotaurine, an intermediate in the biosynthesis of taurine (12). Taurine is an essential osmolyte that maintains keratinocyte hydration, especially in dry environments (13), supporting the skin’s barrier function and overall integrity (14). It also possesses antioxidant properties, protecting human keratinocytes (HaCaT) from ultraviolet light-induced stress and contributing to cell proliferation, membrane stability, inflammation regulation, and collagenogenesis (13, 15). Thus, there are several possible mechanisms for *ADO* to be involved in AD development, and variations in *ADO* expression due to the SNP rs224108 may contribute to AD susceptibility (16).

In this study, we aimed to investigate the role of *ADO* in AD by analysing its function both *in vivo* and *in vitro*. Our analysis showed that the expression of ADO was higher in lesional skin than in normal skin, and immunostaining indicated that *ADO* expression was elevated in lesional skin of AD patients carrying the risk allele of rs10995251 compared with that of non-carriers. We dysregulated *ADO* expression through knocking down and overexpressing it in zebrafish (*Danio rerio*) (17). Analysis of the phenotypic embryos showed that dysregulation of *ADO* resulted in severe abnormalities, particularly in the tail epidermis. Furthermore, altered *ADO* expression in HaCaT cells resulted in modifications in Th2 pathways and the skin barrier.

## MATERIALS AND METHODS

### CRISPR/Cas9-mediated genome editing and functional analysis in zebrafish

The zebrafish orthologues of human *ADO*, *adoa* and *adob*, were targeted using CRISPR/Cas9. Single-guide RNAs (sgRNAs) were co-injected with Cas9 protein into 1-cell embryos, generating mosaic F0 fish. These were outcrossed to produce F1 mutants, phenotyped at 48–72 hpf. For gain-of-function studies, wild-type *adoa*/*adob* mRNAs were synthesized via *in vitro* transcription and injected into embryos. Zebrafish were maintained under standard conditions, including AB WT and *Tg(mpx:GFP)* strains. Genotyping involved HotSHOT DNA extraction, PCR amplification, and Sanger sequencing. Morphological assessment utilized bright-field microscopy, while neutrophil visualization in *Tg(mpx:GFP)* larvae used confocal imaging. Z-stack images of fixed embryos were processed using ImageJ.

### Statistical analysis

All analyses were performed using GraphPad Prism v9.0 (https://www.graphpad.com/). *P*-values ≤ 0.05 were considered statistically significant. Data were expressed as means ± standard deviations (SD). Two-tailed Student’s *t*-tests were performed for the group comparisons. One-way analysis of variance (ANOVA) was used to determine differences among different groups.

## RESULTS

### ADO levels are upregulated in atopic dermatitis lesional skin

**Fig. 1:**
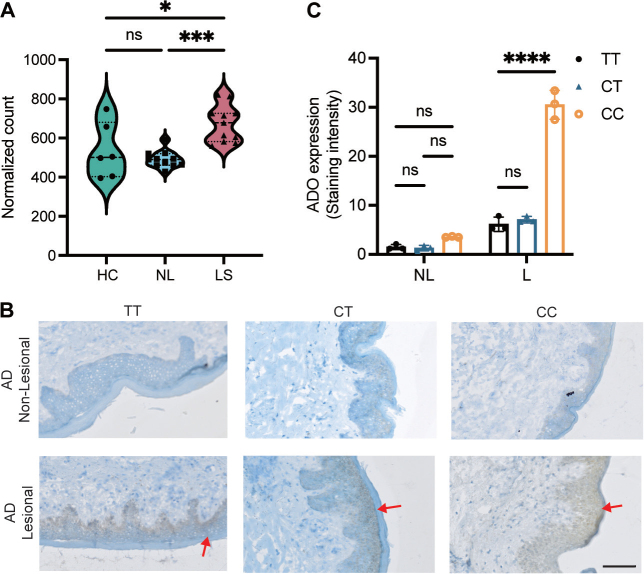
Upregulation of ADO in AD lesional skin in a carrier of the AD-associated risk variant rs10995251. (A) RNA sequencing data from AD lesional skin (LS, *n* = 11) reveal a significant upregulation of ADO compared with non-lesional skin (NL, *n* = 11) and healthy control samples (HC, *n* = 6). Each dot represents a single individual. The data are presented as mean ± standard error of the mean, with statistical significance analysed using ordinary one-way analysis of variance (*p < 0.05, ***p < 0.001). (B) Immunohistochemical analysis of ADO expression in skin biopsies from AD patients stratified by rs10995251 genotype (T/T, C/T, and C/C). Representative images of ADO staining in lesional and non-lesional skin. (C) Quantification reveals significantly higher *ADO* expression in lesional skin (L) of patients carrying the risk allele (C/C and C/T genotypes) compared with the T/T genotype. Statistical analysis was performed using Student’s *t*-test with *p < 0.05, **p < 0.01, ***p < 0.001, and ****p < 0.0001, indicating statistical significance. Scale bars: 100 µm.

In our previous research, using the Capture Hi-C method, we analysed promoter interactions in differentiating keratinocytes. This led to the identification of an interaction between *ADO* promoter and the AD-associated intronic SNP rs10995251, located 166,014 bases away (9). To investigate the expression of the *ADO* gene in lesional skin, we analysed transcriptomic data obtained from the RNA-Seq samples of 11 AD individuals (18). Our findings revealed a significant upregulation of *ADO* expression in AD lesional skin compared with control samples (Fig. 1A), suggesting a possible role of *ADO* in AD pathogenesis. For the AD-associated GWAS variant located on 10q21.2, the C allele has been reported as the risk allele (8, 19). We genotyped Swedish AD patients for the variant rs10995251, identifying 3 distinct geno-types: C/T, C/C, and T/T. To assess whether the risk allele affected ADO expression, we performed immunostaining on skin biopsies from patients with these genotypes. In line with our transcriptomic analysis, ADO expression was markedly elevated in lesional skin compared with non-lesional healthy skin. Furthermore, patients with the risk genotypes (C/C and C/T) had significantly higher ADO expression in lesional skin than those with the T/T genotype (Fig. 1B and C). These results correlate with RNA-Seq data, supporting the conclusion that ADO levels were significantly increased in AD patients.

### ADO orthologues in zebrafish

We used a zebrafish model to validate the candidate gene *ADO* as a contributing factor in AD development. Zebrafish has 2 orthologues of the *ADO* gene: *adoa* and *adob*. Sequence analysis using Clustal (20) revealed a high degree of protein similarity between the zebrafish orthologues and human ADO, indicating a potential conservation of function across species (Figs S1–3). This conservation suggests that zebrafish may serve as a relevant model to study the function and impact of *ADO* in inflammatory conditions.

### CRISPR/Cas9-mediated knockout and overexpression of adoa and adob alter epidermal morphogenesis

**Fig. 2:**
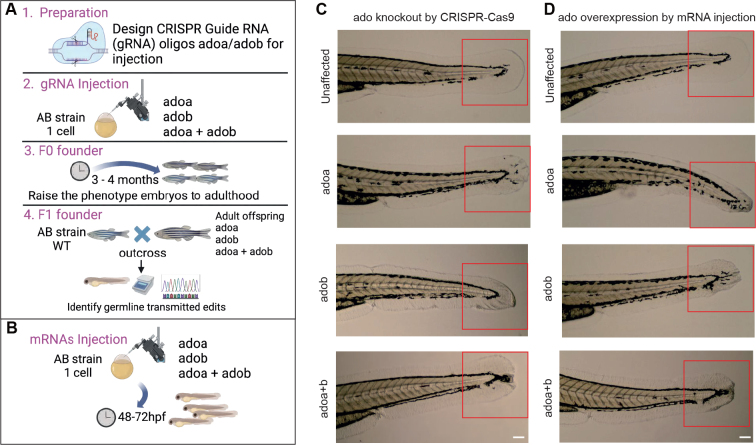
Dysregulated *ADO* levels lead to epidermal disruption in zebrafish tails. (A–B) Schematic representation of the experimental design for CRISPR/Cas9-mediated knockout of *adoa* and *adob* in zebrafish embryos. Guide RNAs targeting *adoa* and *adob* genes were injected into wildtype (WT, AB strain) zebrafish embryos, generating mosaic F0 embryos. The injected embryos were raised to adulthood. Gain-of-function experiments involved the injection of 400 ng wildtype *adoa* and *adob* mRNAs into 1–2-cell stage zebrafish embryos. CRISPR/Cas9 editing on embryos was confirmed by Sanger sequencing, with a shorter band being amplified as a result of the deletion. The image was created with BioRender.com. hpf: hours post-fertilization. (C) Representative 10 x images of zebrafish embryos displaying epidermal abnormalities. Heterozygous loss of *ADO* expression resulted in severe morphological defects at 72 hpf, particularly in the tail fin, which consists solely of epidermal cells. (D) Overexpression, achieved by injection of 400 ng *adoa* and *adob* mRNAs into 0–1-cell stage zebrafish embryos, also revealed notable abnormalities at 72 hpf. Scale bars: 100 µm.

First, to determine the impact of *adoa* and *adob* on zebrafish epidermal development, we used a loss-of-function approach involving CRISPR/Cas9 gene editing (Fig. 2A). In parallel, to model the gene overexpression observed in human AD patients, we conducted gain-of-function experiments by injecting wild-type (WT) *adoa* and *adob* mRNAs into zebrafish embryos (Fig. 2B).

In the second-generation offspring of the *adoa* and *adob* knockout zebrafish, the caudal fin exhibited impaired formation, characterized by reduced length and frequent buckling towards the tail end. Additionally, the epidermis appeared disorganized and incomplete, with ragged edges observed on both the ventral and dorsal sides (Fig. 2C). Interestingly, embryos overexpressing *adoa* and *adob* displayed phenotypes strikingly similar to those observed in the knockout experiments. The caudal fin was consistently shorter compared with non-phenotypic embryos and the epidermis showed signs of improper formation with buckling noted at the tail end (Fig. 2D). Collectively, these results demonstrated that both knockout and overexpression of *adoa* and *adob* significantly disrupted epidermal development in zebrafish, indicating a crucial role for these genes in proper epidermis formation.

### Dysregulated levels of ADO lead to enhanced neutrophil recruitment in zebrafish skin

Neutrophils are key components of the innate immune system, acting as first responders to tissue inflammation. Their role is well established in other inflammatory skin disorders, such as psoriasis. Recent studies have also shown potential roles of neutrophils in certain types of AD, though their exact function remains unclear (21). In zebrafish, the innate immune system becomes fully functional at 24–48 hpf, facilitating the recruitment of neutrophils to sites of injury or infection (22). Skin-infiltrating neutrophils are implicated in conditions such as AD (23), and we hypothesized that abnormal levels of *ADO* might alter neutrophil activity, contributing to the skin abnormalities observed in certain zebrafish phenotypes.

**Fig. 3:**
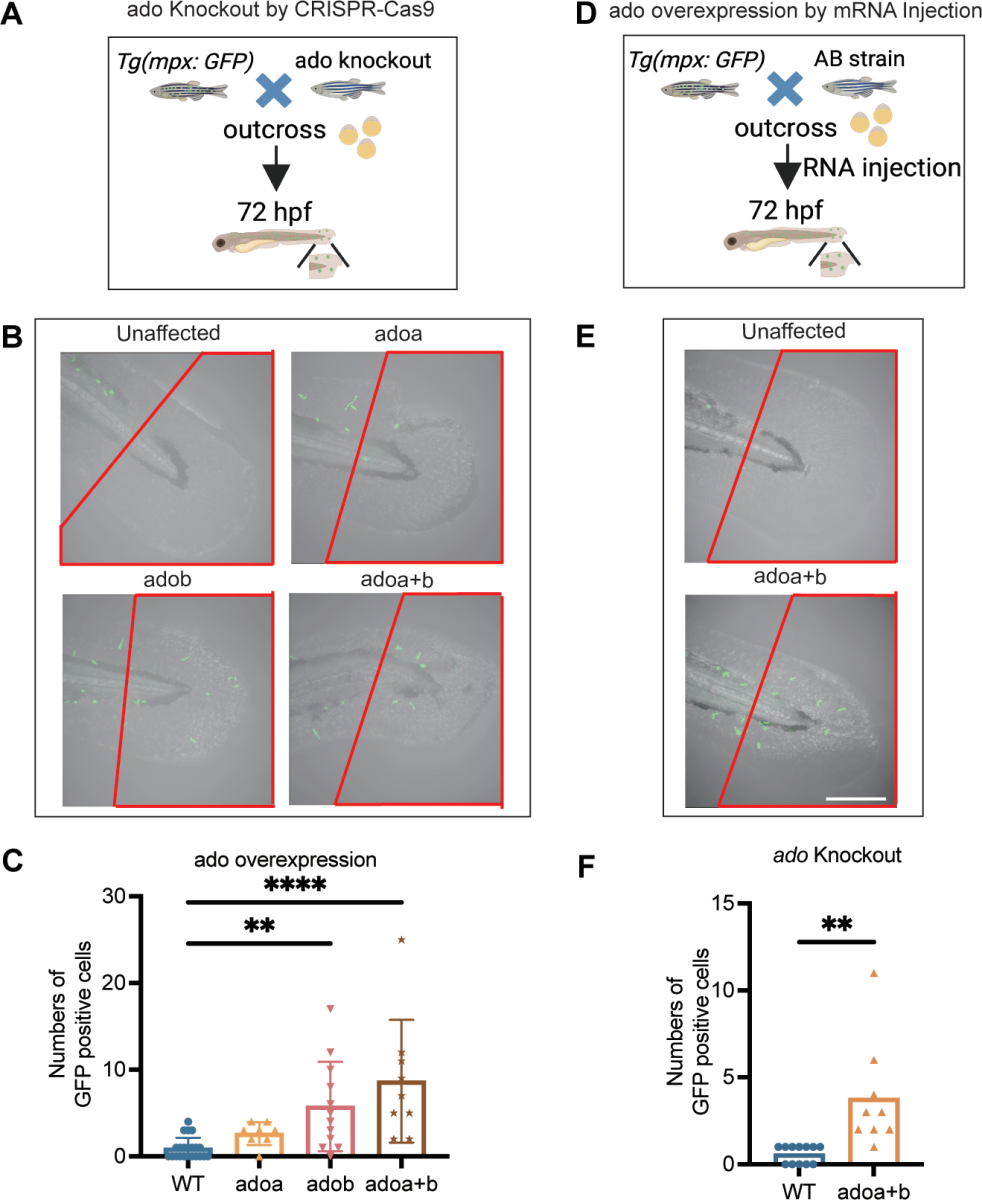
Dysregulated *ADO* levels promote enhanced neutrophil recruitment. (A) Schematic illustration of the experimental approach to study neutrophil recruitment in zebrafish embryos. Transgenic *Tg*(*mpx*) zebrafish, expressing green fluorescent protein (GFP) in neutrophils, were used. The *ADO* overexpression was induced by injecting 400 ng of *ADO* mRNA into 1–2-cell stage *Tg*(*mpx*) embryos. The image was created with BioRender.com. hpf: hours post-fertilization. (B–C) Representative fluorescent images of *Tg*(*mpx*) embryos at 72 hpf following *ADO* knockdown. Neutrophils were visualized and quantified in controls and *ADO* knockout embryos with quantification performed in the outlined areas. (wild-type (WT), *n* = 22; *adoa n* = 8; *adob*, *n* = 12, *p* = 0.0027; *adoa+b*, *n* = 9). (D) Schematic illustration of the experimental approach to study neutrophil recruitment in zebrafish embryos. *Tg*(*mpx*) zebrafish were outcrossed with CRISPR/Cas9-mediated *ADO* knockout zebrafish. (E–F) Representative fluorescent images in *Tg*(*mpx*) embryos at 72 hpf following *ADO* overexpression. Neutrophils were visualized and quantified in the *ADO*-overexpressing embryos with quantification performed in the outlined areas (WT, *n* = 12; *adoa+b*, *n* = 9, scale bars = 100 μm). Data are presented as mean ± standard error of the mean, with statistical significance indicated. (**p < 0.01, ****p < 0.0001).

To evaluate how immune cells, particularly neutrophils, respond to altered skin conditions, we utilized a transgenic zebrafish line *Tg(mpx: GFP)*, where neutrophils were fluorescently labelled with green fluorescent protein (GFP). We outcrossed this transgenic line with zebrafish that had the *ADO* gene knocked out (Fig. 3A). Our analysis revealed a significant increase in GFP-positive neutrophils in the tails of phenotypic embryos (WT, *n* = 22; *adoa*, *n* = 8; *adob*, *n* = 12, *p* = 0.0027; *adoa+b*, *n* = 9, *p* < 0.0001). This suggests that downregulation of the *ADO* gene is associated with an increase in neutrophil recruitment, which may contribute to the skin abnormalities observed in these embryos (Fig. 3B and C).

Further investigation was conducted to understand the effects of *ADO* gene overexpression. *ADO* mRNA (400 ng) was injected into *Tg(mpx: GFP)* embryos (Fig. 3D). These results resembled those seen in the knockout models, with a significant increase in the number of neutrophils found outside the blood vessels in embryos overexpressing the *ADO* gene (WT, *n* = 12; *adoa+b*, *n* = 9, *p* < 0.0001) (Fig. 3E and F), indicating that both loss and overexpression of *ADO* can lead to abnormal neutrophil activity. In conclusion, our study provides strong evidence that proper *ADO* gene function is essential for normal skin development, with neutrophil recruitment playing a crucial role in the observed phenotypic changes.

### ADO disrupts pro-inflammatory cytokines secretion and skin barrier integrity in HaCaT cells

**Fig. 4:**
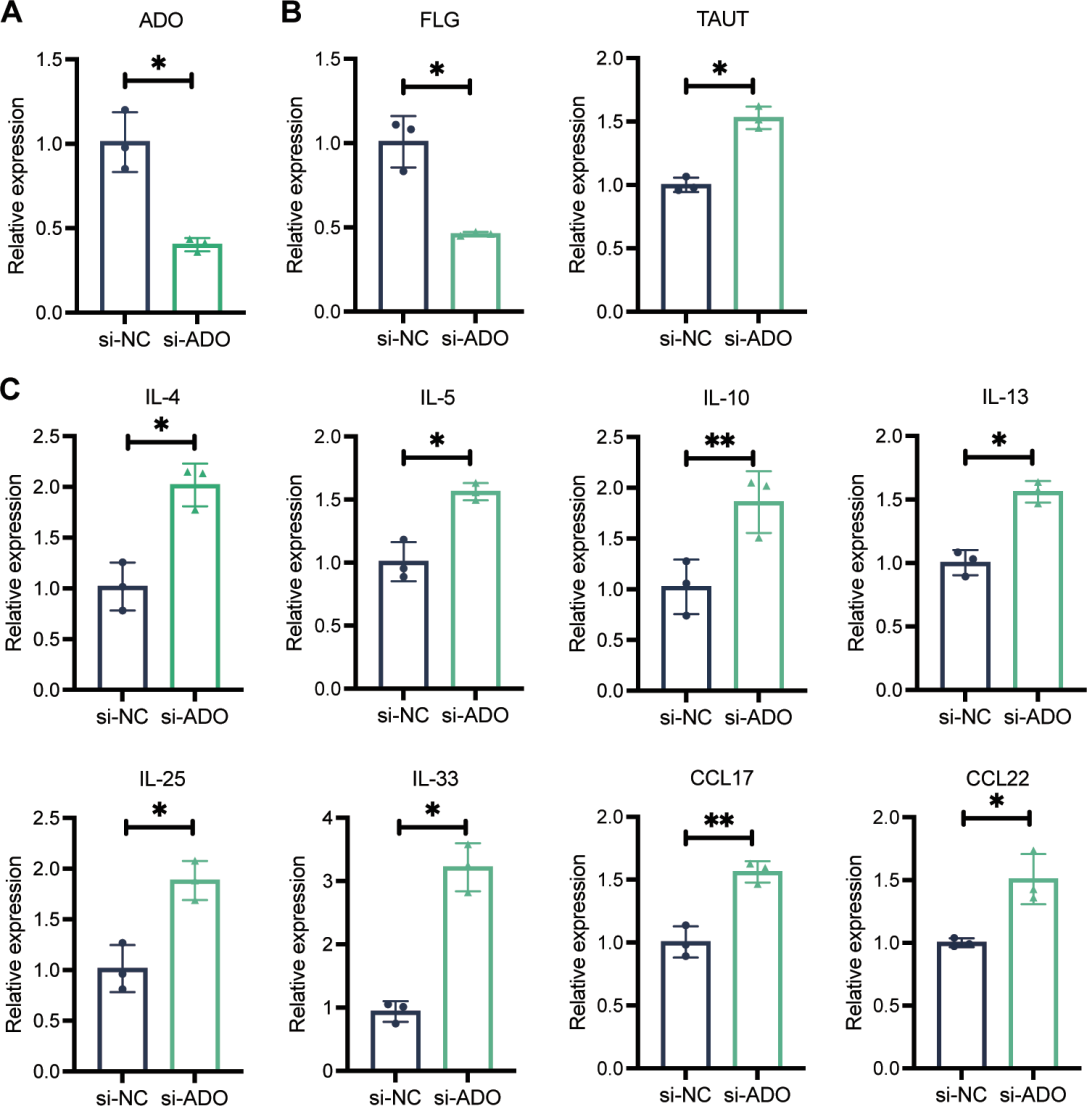
Impact of *si-ADO* on inflammatory and immune cytokine expression in HaCaT cells. (A) Transfection with *si-ADO* in HaCaT cells significantly reduced *ADO* mRNA expression levels. (B) The mRNA expression of *FLG*, which encodes filaggrin, an essential protein for skin barrier function, was significantly downregulated in response to *ADO* knockdown. *ADO* knockdown also resulted in increased mRNA levels of taurine transporter (*TAUT*), suggesting a potential compensatory mechanism aimed at maintaining skin barrier integrity. (C) Cytokine profiling of *ADO*-silenced cells showed the elevations in pro-inflammatory cytokines, including *IL*-*4*, *IL-5*, *IL-10*, *IL-13*, IL-25, *IL-33*, *CCL17*, and *CCL22* (*p* < 0.001), highlighting the role of *ADO* in promoting inflammation in HaCaT cells. Statistical significance was determined using Student’s *t*-test. *p < 0.05 and **p < 0.01.

To study the role of *ADO* in HaCaT cells, we utilized ADO-specific small interfering RNA (siRNA) for transient transfections. Compared with the control group (*si-NC*), the *si-ADO* group had a significantly lower relative expression of *ADO* based on the results of qRT-PCR (*p* < 0.05, Fig. 4A), indicating successful knockdown. FLG is a key protein for maintaining skin barrier integrity, and mutations in the *FLG* gene are a major risk factor for AD (24). *FLG* expression was found to be lower in the *si-ADO* group (*p* < 0.05, Fig. 4B). The taurine transporter (TAUT) is involved in regulating skin hydration and protecting against oxidative stress, which indirectly affects skin health in AD (13). Our results indicated that *TAUT* levels were significantly higher in the *si-ADO* group (*p* < 0.05, Fig. 4B), suggesting a potential compensatory response. In addition to skin epidermal barrier changes, our results showed that the mRNA levels of interleukin 4 (*IL4*), *IL-5*, *IL-13*, *IL-25*, *IL-33*, *CCL17*, and *CCL22* were significantly increased in HaCaT cells where *ADO* expression was silenced (*p* < 0.05, Fig. 4C). This suggests that *ADO* knockdown enhances the expression of proinflammatory cytokines and chemokines involved in AD pathogenesis.

**Fig. 5:**
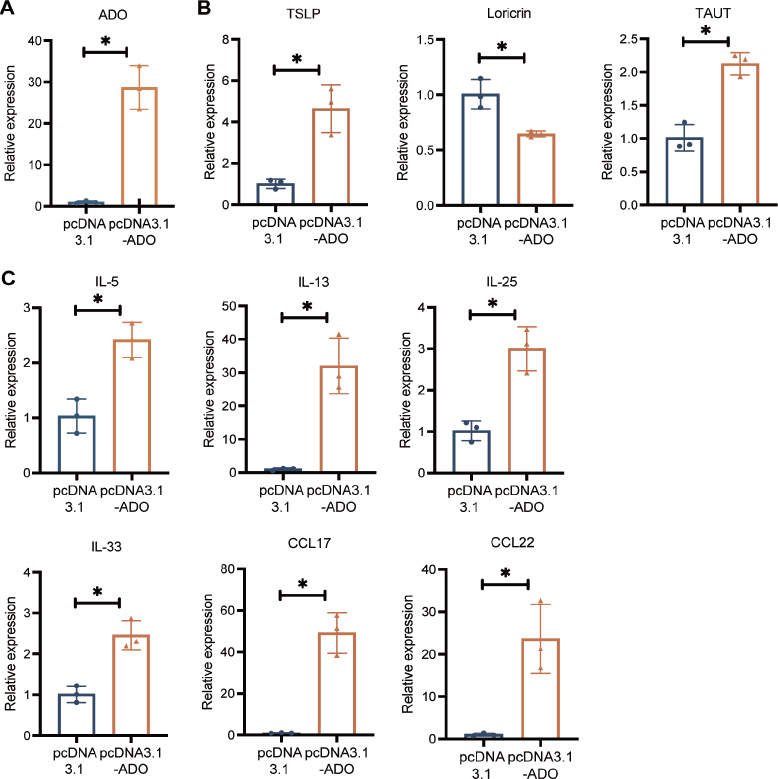
Effects of inflammatory and immune cytokines on upregulation of *ADO* in HaCaT cells. (A) *ADO* expression was assessed through qRT-PCR in HaCaT cells stably transfected with pcDNA3.1-ADO-GFP. GAPDH was used as a loading control. Relative *ADO* levels were quantified from 3 independent experiments. (B) *Loricrin* expression was significantly decreased, potentially contributing to impaired skin barrier. *ADO* overexpression led to increased mRNA levels of *TAUT*, and thymic stromal lymphopoietin (TSLP) suggesting a potential compensatory mechanism to maintain skin barrier integrity. (C) Cytokine expression analysis in *ADO*-overexpressing HaCaT cells showed significant upregulation of proinflammatory cytokines and chemokines, including *IL-5*, *IL-13*, *IL-25*, *IL-33*, *CCL17*, *CCL22*. These results suggest an enhanced inflammatory response associated with *ADO* overexpression. Statistical significance was determined using Student’s *t*-test. *p < 0.05.

To further explore the functional relevance of *ADO* upregulation in AD, we generated HaCaT lines stably overexpressing *ADO* by transfecting them with pcDNA3.1(+)-ADO-GFP. The results showed a 28-fold increase in *ADO* mRNA levels in the transfected cells compared with controls (Fig. 5A).

Notably, the overexpression of *ADO* led to an increase in thymic stromal lymphopoietin (TSLP), a cytokine known to activate dendritic cells and promote Th2 immune responses (Fig. 5B). TSLP is often associated with the initiation and exacerbation of AD, further supporting the role of *ADO* in driving inflammatory processes. Loricrin, a crucial structural protein in the epidermal barrier, was significantly decreased, whereas *TAUT* expression was elevated, possibly as a protective response to maintain skin homeostasis (Fig. 5B). In *ADO*-overexpressing HaCaT cells, pro-inflammatory cytokines such as *IL-5*, *IL-13*, *IL-25*, *IL-33*, *CCL17*, and *CCL22* were markedly elevated (Fig. 5C).

Overall, these results suggest that dysregulation of *ADO* in HaCaT cells not only induces secretion of proinflammatory cytokines but also perturbs key components of the skin barrier and immune response.

### Involvement of reactive oxygen species levels is higher when ADO is downregulated

**Fig. 6:**
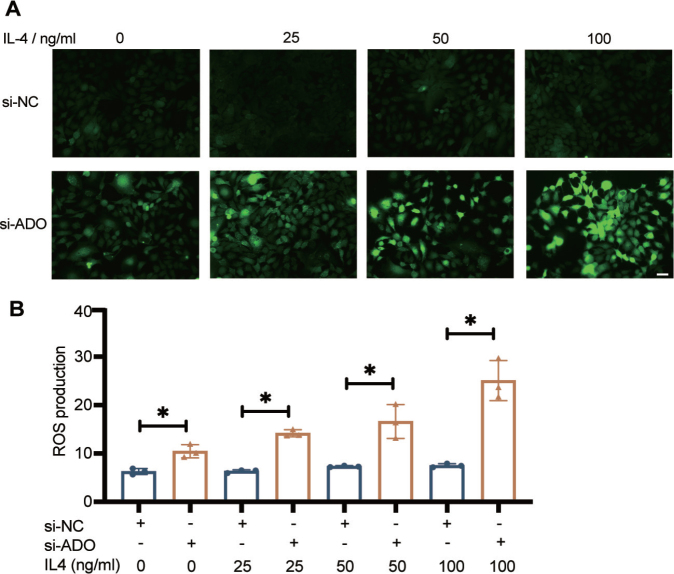
Role of ROS in IL4-induced downregulation of *ADO* in HaCaT cells. (A) Representative immunofluorescence images showing reactive oxygen species (ROS) levels in HaCaT cells. ROS levels were measured using 10 μM DCFH-DA for 30 min. HaCaT cells were stimulated with varying concentrations of IL-4 (0, 25, 50, and 100 ng/mL) to model AD for 12 h before ROS detection. Scale bar: 100 μm. (B) Quantification of mean fluorescence intensity of ROS using ImageJ. The *si-ADO* cells exhibited significantly higher ROS generation than si-NC controls. Student’s *t*-test was used to detect statistical significance. *p < 0.05.

Interactions between Th2-type inflammation and skin barrier defects lead to chronic AD inflammation. Chronic skin inflammation, pruritus, and skin microbiome are linked to overproduction of reactive oxygen species (ROS) (25). Previous studies have shown that depleted *ADO* in liver cancer cells results in high levels of ROS and an increased ratio of oxidized to reduced glutathione, which is indicative of oxidative stress (26). In our study, the dichlorodihydrofluorescein diacetate (DCFH-DA) assay revealed significantly higher ROS production in HaCaT cells when *ADO* expression was knocked down (**Fig. 6**A and B). ROS are known to exacerbate inflammation by activating various signalling pathways that lead to the production of proinflammatory cytokines such as IL-4 (27). To further explore the relationship between IL-4 and ROS, HaCaT cells were treated with 25, 50, and 100 ng/µL of human IL-4 recombinant protein for 24 h. We observed a marked increase in ROS fluorescence intensity in IL-4-treated cells compared with untreated cells (Fig. 6A and B), indicating that IL-4 not only participates in the Th2 pathway but also promotes oxidative stress by enhancing ROS production.

## DISCUSSION

Our study provides a comprehensive analysis of *ADO* in AD by integrating transcriptomic data, GWAS, and functional experiments in both zebrafish and HaCaT lines. We present evidence that *ADO* is significantly upregulated in lesional skin of AD patients, correlated with the AD-associated SNP rs10995251, and modulates skin inflammation and barrier integrity. The upregulation of *ADO* in AD lesional skin was demonstrated through RNA-Seq analysis and supported through immunostaining.

Interestingly, the SNP rs10995251, located on chromosome 10q21.2, has been implicated in susceptibility to AD (9). This SNP is also associated with the *ZNF365* gene, which is located in the same chromosomal region, as reported in the GWAS (28). Our findings reinforce the hypothesis that the rs10995251 variant can modulate *ADO* gene expression and contribute to disease pathogenesis. This is further supported by independent GWAS analyses that have identified *ADO* as a candidate gene for AD (5, 8).

Our study revealed that dysregulation of *ADO* resulted in Th2 secretion in HaCaT cells. Th2 cells have an important role in the pathophysiology of AD. IL-4 and IL-13 contribute to allergic inflammation, influence the skin microbiome, and impair epidermis barrier function (1). IL-5, another important cytokine, is crucial for the recruitment of eosinophils to site of allergen exposure (29). IL-10, IL-25, and IL-33 are epithelial cell-derived cytokines that promote immune responses, particularly through the activation of innate lymphoid cells and eosinophils (3). CCL17 and CCL22 are key drivers of allergic inflammation in AD (30).

*ADO* is recognized as a key human enzymatic oxygen sensor, responsible for catalysing the oxidation of N-terminal cysteine in proteins, much like the plant cysteine oxidases that mediate hypoxia responses. It regulates protein oxygen stability by controlling the degradation of substrates for the G-protein signalling regulators RGS4/5 via N-terminal modification (31). The hypoxic microenvironment, characteristic of AD lesions, increases immune cell recruitment, drives pro-inflammatory cytokine release, and impairs keratinocyte differentiation – all processes that are dysregulated in AD (32).

Additionally, hypoxia contributes to increased oxidative stress through higher levels of ROS, a key feature of AD pathogenesis (33). Our data show that the dysregulation of *ADO* expression leads to elevated ROS levels, potentially leading to the chronic inflammatory state observed in AD. It has been reported that elevated ROS levels can damage skin, exacerbate inflammation, and ultimately worsen AD symptoms (25). Moreover, ROS can activate downstream pathways such as nuclear factor kappa B and transcription factor activator protein -1, known regulators of inflammatory cytokine production (34).

Hypoxia-inducible factor 1-alpha may also play a role in perpetuating skin inflammation by driving the expression of proinflammatory mediators under hypoxic conditions (35). Furthermore, recent evidence suggests that *ADO*-mediated taurine regulation impacts mitochondrial metabolism in conditions such as obesity and metabolic diseases (36), as well as in glioblastoma (12) and malignant plasma cells (37). In systemic inflammatory disorders, *ADO* has been linked to *ZNF365* and *EGR2* as part of a novel genetic risk locus associated with Vogt–Koyanagi–Harada disease. This is a polymorphic inflammatory disorder that targets organs with melanocytes, including the skin, eyes, and meninges (38). These findings further highlight *ADO*’s involvement in broader immunological and inflammatory processes, beyond its role in AD.

The zebrafish is a well-established model system widely used in various research fields, including drug discovery and cancer biology (39). Over the past 2 decades, zebrafish have emerged as a valuable model for dermatological diseases such as vitiligo, psoriasis, and wound healing (17), due to similarities with human skin. Additionally, the zebrafish model allows for *in vivo* studies of uncharacterized genes and variants, offering deeper insights into their roles compared with *in vitro* models (40). However, despite its advantages, the zebrafish is not a mammal, and there are still significant morphological and biological differences that limit its ability to fully replicate human skin conditions. Nevertheless, it remains a powerful tool for addressing specific research questions and exploring gene function in the context of skin biology.

Our study has several limitations. Although the use of the zebrafish model and HaCaT cells offered valuable insights into *ADO*’s role, these systems may not fully capture the complexity of human skin and AD pathophysiology. Moreover, although we observed increased ROS production and altered cytokine profiles in response to *ADO* dysregulation, the precise molecular mechanisms driving these changes remain incompletely understood.

In conclusion, our findings establish *ADO* as a crucial player in the pathogenesis of AD, underscoring its dual role in modulating immune responses and maintaining skin barrier integrity. Our study not only highlights the genetic underpinnings of *ADO*’s involvement but also provides a foundation for further exploration of its mechanistic role in AD. Future investigations should focus on unravelling the intricate signalling networks that *ADO* modulates, with the ultimate goal of developing novel therapeutic interventions aimed at alleviating AD symptoms. By targeting *ADO*, it may be possible to reduce inflammation and protect the skin barrier.

## Supplementary Material




