# Supplementary material for: The Role of the Cysteamine Dioxygenase (ADO) Gene in Atopic Dermatitis
Source: Acta Derm Venereol. 2026 Jan 27;106:43770. doi: 10.2340/actadv.v106.43770 (PMC12856578; doi:10.2340/actadv.v106.43770)
Supplement: Supplementary file 1 [file ActaDV-106-43770-s1.pdf]

Supplementary material has been published as submitted. It has not been copyedited, or typeset by Acta Dermato-Venereologica

Fig. S1. Alignment of the amino acid sequences of the human ADO and the zebrafish orthologues Adoa. The human ADO shares 54.1 % similarity with the zebrafish Adoa. >NP_116193.2 2-aminoethanethiol dioxygenase [Homo sapiens], >NP_001008634.1 2-aminoethanethiol (cysteamine) dioxygenase a [Danio rerio].

```
CLUSTAL O(1.2.4) MULTIPLE SEQUENCE ALIGNMENT


NP_116193.2         MPRDNMASLIQRIARQACLTFRGSGGGRGASDRDAASGPEAPMQPGFPENLSKLKSLLTQ        60
NP_001008634.1      MPRNNKTSLIQKIASQAYATFTNCSSSAIE-----------DNNKVFLEHQADLVALLSD        49
                    ***:* :****:** **  ** .....                :  * *: :.* :**::

NP_116193.2         LRAEDLNIAPRKATLQPLPPNLPPVTYMHIYETDGFSLGVFLLKSGTSIPLHDHPGMHGM        120
NP_001008634.1      IRAADLKIAPPTK-VSTSSPSVPPVTYMHICETDVFSMGVFLLKSGASIPLHDHPGMHGM        108
                    :** **:*** .  :.   *.:******** *** **:********:*************

NP_116193.2         LKVLYGTVRISCMDKLDAGGGQRPRALPPEQQFEPPLQPREREAVRPGVLRSRAEYTEAS        180
NP_001008634.1      LKVLYGKVSIRCYDKLDKAESD------TERHFDPPLLAFQGDDVRRAALRSSGQFSELS        162
                    ******.* * * **** . .:       *::*:***   : : ** ..*** .:::* *

NP_116193.2         GPCILTPHRDNLHQIDAVEGPAAFLDILAPPYDPDDGRDCHYYRVLEPVRPKEASSSACD        240
NP_001008634.1      GPCVLSPFKDNLHEIDAVDGAAAFLDILAPPYDPDDGRDCHYYRVLQTAGKKSEQ----S        218
                    ***:*:*.:****:****:* *************************: .  *. .    .

NP_116193.2         LPREVWLLETPQADDFWCEGEPYPGPKVFP      270
NP_001008634.1      GDDEAWLLEIPQPDDFWCGGEPYPGPEVTI      248
                       *.**** ** ***** *******:*
```

Fig. S2. Alignment of the amino acid sequences of the human ADO and the zebrafish orthologues Adob. The human ADO shares 54.4 % similarity with Adob. >NP_998358.2 2-aminoethanethiol (cysteamine) dioxygenase b [Danio rerio].

```
CLUSTAL O(1.2.4) MULTIPLE SEQUENCE ALIGNMENT


NP_116193.2      MPRDNMASLIQRIARQACLTFRGSGGGRGASDRDAASGPEAPMQPGFPENLSKLKSLLTQ   60
NP_998358.2      MPRDNMTSTVQKIAKQALATFRNPSVI-------------GEHNKVFLENQSKLKSLLAE   47
                 ******:* :*:**:**  ***. .               .  :  * ** *******::

NP_116193.2      LRAEDLNIAPRKATLQPLPP--NLPPVTYMHIYETDGFSLGVFLLKSGTSIPLHDHPGMH   118
NP_998358.2      VRAADLKIAARTPESAPVPHQRIAPPVTYMHICETDSFSMGVFLLKTGASIPLHDHPGMY   107
                 :** **:** *.    *:*     ******** ***.**:******:*:**********:

NP_116193.2      GMLKVLYGTVRISCMDKLDAGGGQRPRALPPEQQFEPPLQPREREAVRPGVLRSRAEYTE   178
NP_998358.2      GMLKVIYGKVRISCFDRLDK-----PRDGASGVQFNPPLMPFQRGSLRPSVLKSVGEFTE   162
                 *****:**.*****:*:**      **      **:*** * :* ::**.**:* .*:**

NP_116193.2      ASGPCILTPHRDNLHQIDAVEGPAAFLDILAPPYDPDDGRDCHYYRVLEPVRPKEASSSA   238
NP_998358.2      DSSPCVLSPQQDNIHQIDAVDGPTAFLDILAPPYDPDEGRDCHYYKVLQAHSEAADKKSE   222
                  *.**:*:*::**:******:**:*************:*******:**:       ..*

NP_116193.2      CDLPREVWLLETPQADDFWCEGEPYPGPKVFP       270
NP_998358.2      VQDQGDVWLMEIPQPSEFWCGGEPYPGPKVTL       254
                  :   :***:* ** .:*** *********
```

Fig. S3. Alignment of the amino acid sequences of the human ADO, the zebrafish orthologues Adoa, and the zebrafish orthologues Adob.

The two orthologues share 56.3 % identity between them.

```
CLUSTAL O(1.2.4) MULTIPLE SEQUENCE ALIGNMENT


NP_116193.2         MPRDNMASLIQRIARQACLTFRGSGGGRGASDRDAASGPEAPMQPGFPENLSKLKSLLTQ        60
NP_001008634.1      MPRNNKTSLIQKIASQAYATFTNCSSSAI-----------EDNNKVFLEHQADLVALLSD        49
NP_998358.2         MPRDNMTSTVQKIAKQALATFRNP--SVI-----------GEHNKVFLENQSKLKSLLAE        47
                    ***:* :* :*:** **  ** .   .                :  * *: :.* :**::

NP_116193.2         LRAEDLNIAPRKATLQPL--PPNLPPVTYMHIYETDGFSLGVFLLKSGTSIPLHDHPGMH        118
NP_001008634.1      IRAADLKIAPPTKVSTS---SPSVPPVTYMHICETDVFSMGVFLLKSGASIPLHDHPGMH        106
NP_998358.2         VRAADLKIAARTPESAPVPHQRIAPPVTYMHICETDSFSMGVFLLKTGASIPLHDHPGMY        107
                    :** **:**  .            ******** *** **:******:*:**********:

NP_116193.2         GMLKVLYGTVRISCMDKLDAGGGQRPRALPPEQQFEPPLQPREREAVRPGVLRSRAEYTE        178
NP_001008634.1      GMLKVLYGKVSIRCYDKLDKA-----ESD-TERHFDPPLLAFQGDDVRRAALRSSGQFSE        160
NP_998358.2         GMLKVIYGKVRISCFDRLDKP-----RDGASGVQFNPPLMPFQRGSLRPSVLKSVGEFTE        162
                    *****:**.* * * *:**       .      :*:***   :   :* ..*:* .:::*

NP_116193.2         ASGPCILTPHRDNLHQIDAVEGPAAFLDILAPPYDPDDGRDCHYYRVLEPVRPKEASSSA        238
NP_001008634.1      LSGPCVLSPFKDNLHEIDAVDGAAAFLDILAPPYDPDDGRDCHYYRVLQTAG----KKSE        216
NP_998358.2         DSSPCVLSPQQDNIHQIDAVDGPTAFLDILAPPYDPDEGRDCHYYKVLQAHSEAADKKSE        222
                     *.**:*:* :**:*:****:* :*************:*******:**:       ..* 

NP_116193.2         CDLPREVWLLETPQADDFWCEGEPYPGPKVFP    270
NP_001008634.1      QSGDDEAWLLEIPQPDDFWCGGEPYPGPEVTI    248
NP_998358.2         VQDQGDVWLMEIPQPSEFWCGGEPYPGPKVTL    254
                     .   :.**:* ** .:*** *******:*
```
