# Supplementary material for: The Role of the Cysteamine Dioxygenase (ADO) Gene in Atopic Dermatitis
Source: Acta Derm Venereol. 2026 Jan 27;106:43770. doi: 10.2340/actadv.v106.43770 (PMC12856578; doi:10.2340/actadv.v106.43770)
Supplement: Supplementary file 2 [file ActaDV-106-43770-s2.pdf]

Supplementary material has been published as submitted. It has not been copyedited, or typeset by Acta Dermato-Venereologica

Table SI. sgRNAs used for CRISPR/Cas9 genome editing.

**gene**	**location**	**sgRNA sequence**
adoa	-500	GGCACTGTTGCATTTGTGTCAGC
	+500	CCAACAATGGAGGATCAAAGTGCC
adob	-500	GTGGTGTTTGTGATTGTTCATACAG
	+500	CCCTCTGGAAGGGCATGAGCGG

sgRNA sequences used to knock out adoa/b and their distance to the ATG start codon (location, in nucleotides). sgRNA: single guide RNA.

Table SII. Primers used for genotyping of *adoa* and *adob*.

**gene**	**directio n**	**Primer Sequence**
adoa	forward	CCGAGGAACAACAAGACCTCGCTC
	reverse	CAGCCCTGATGTCGGAGAGCAGGG
adob	forward	GCCACGAGACAACATGACTTCC
	reverse	GCCGCTCTGACCTCCGCCAAGAG

Table SIII. Primers used for RT-qPCR.

Target gene	Forward	Reverse
ACTB	CAACCGCGAGAAGATGAC	AGGAAGGCTGGAAGAGTG
ADO	CGAGAACCTGAGCAAGCTGAAG	GTCCGTCTCGTAGATGTGCATG
IL-4	CCACGGACACAAGTGCGATA	CCCTGCAGAAGGTTTCCTTCT
IL-5	TCTACTCATCGAACTCTGCTG	CCCTTGCACAGTTTGACTCTC
IL-10	GTGATGCCCCAAGCTGAGA	CACGGCCTTGCTCTTGTTTT
IL-13	TGTTTGTCACCGTTGGGGAT	TGAGTCTCTGAACCCTTGGC
IL-33	TCCCAACAGAAGGCCAAAGA	AAAGGCAAAGCACTCCACAG
IL-25	TGTACCAGGTCAGTGCAGAG	GTTCCCATGACCATTGCCAA
CCL17	CGGACCCCAACAACAAGAGA	CTCCCTCACTGTGGCTCTTC
CCL22	GAAGCCTGTGCCAACTCTCT	GGGAATCGCTGATGGGAACA
FLG	TGAAGCCTATGACACCACTGA	TCCCCTACGCTTTCTTGTCCT
Taut	CTGCCCCTTGTTCTCTGGTA	TTGGTGGAGCTGATGGTGAT
Loricin	GGCCGTCCAAATAGATCCCC	AACTAGATGCAGCCGGAGAG
TSLP	ACAACTTGTAGGGCTGGTGT	AACATTTCTTTGGCGAGCGA
FLG2	GCAACAAGGTCCTCAGCAAA	CCCTTGAGTGCCCAGAACTA
